# Gender empowerment and environmental impact: A quantile regression analysis in BRICS economies

**DOI:** 10.1016/j.heliyon.2024.e40056

**Published:** 2024-11-06

**Authors:** Fortune Ganda

**Affiliations:** Faculty of Economic and Financial Sciences, Department of Management Accounting and Finance, Walter Sisulu University, Zamukulungisa Campus, Private Bag X1, Mthatha, 5117, Mthatha, South Africa

**Keywords:** Women's political empowerment index, Women's civil liberties index, Women as government chief executives, Carbon emissions, Economic growth, Population growth

## Abstract

This study, with its significant findings, delves into the impact of women's status on environmental quality in BRICS economies (Brazil, Russia, India, China, South Africa) from 1960 to 2022. Using a novel method, the Method of Moments Quantile Regression, this study has been able to analyse the relationship. The results, which are of utmost importance, show that women's political empowerment and leadership positions (government chief executives) significantly reduce carbon emissions, while the impact of women's civil liberties along with population growth increases emissions across the analysed quantiles. Economic growth is insignificantly negatively associated with environmental quality. The paper's findings reveal a unidirectional causal relationship from women's political empowerment to emissions, carbon emissions to women's civil liberties and emissions to economic growth. Additionally, a bi-directional causality connection is evident between population growth and environmental quality. These insights, which are crucial for policymaking, suggest that promoting improved women's status is a crucial policy strategy for mitigating climate change in BRICS economies. Thus, this paper suggests that empowerment of women is an effective strategy for reducing carbon emissions. It emphasizes the need for climate policy to promote gender equality, prioritise women's leadership in the clean energy industry, and enhance their access to resources and opportunities.

## Highlights

• BRICS nations witness a dramatic drop in carbon emissions with increased female status.
• Women's political empowerment develop a statistically significant negative relationship with carbon emissions.
• Women's political participation drives down carbon emissions.
• Women in top leadership positions (government chief executives) significantly curb carbon emissions, while women's civil liberties considerably heighten emissions.
• Unidirectional causality from women's political empowerment to emissions, carbon emissions to women's civil liberties and emissions to economic growth is demonstrated. The bi-directional causality connection between population growth and environmental quality is evident.

## 1 Introduction

The adverse impacts of climate change might manifest in the immediate term through occurrences of natural hazards, such as landslides, floods, and storms. Additionally, over an extended period, the natural environment will experience a steady deterioration as a result of climate change [1,2]. The negative consequences of these occurrences are already being experienced in various domains, such as agriculture and food security, biodiversity and ecosystems, water resources, human health, human settlements and migratory patterns, and energy, transport, and industry [[3], [4], [5], [6]]. As such, it is imperative to incorporate women, girls, and marginalised populations who are disproportionately impacted by climate change in the planning and execution of climate response measures [7]. This inclusive approach is necessary to ensure equitable distribution of benefits.

There is evidence which points out that women possess distinctive knowledge and experience; therefore, their participation in decision-making processes is essential for effective climate action [8]. For instance, female participation in national parliaments has been observed to correlate with the implementation of more rigorous climate change legislation, therefore leading to a reduction in emissions [9]. The involvement of women in natural resource management at the local and national levels has been found to be associated with enhanced resource governance and conservation outcomes. To boot, the presence of women in leadership positions within the workplace has been found to be linked with a higher level of transparency regarding the impact of climate change [10]. There exists a favourable correlation between higher proportions of women serving on boards of companies and the publication of carbon emissions information [11].

Regrettably, in numerous instances, women are found to be more susceptible to the impacts of climate change compared to men [12]. This is primarily due to their higher representation among the global impoverished population and their greater reliance on natural resources, which are at risk due to climate change. Moreover, women encounter various social, economic, and political obstacles that restrict their ability to effectively manage and adapt to challenges [13]. Women residing in rural regions inside emerging nations face heightened vulnerability since the majority rely heavily on local natural resources for their sustenance. This paper examines how the status of women influences environmental quality in emerging economies, widely understood as the BRICS countries.

[14] laments that the BRICS are among the top 10 highest global emitters, and, hence, vulnerable groups, such as women, migrant workers, older individuals, individuals in poverty and persons with disabilities, are disproportionately impacted by the risks and hazards stemming from environmental degradation. These effects vary depending on the country or region, ultimately leading to the creation and perpetuation of inequality [15]. expose that the persistence of gender disparities in various social and economic domains is a well-documented phenomenon, particularly within the field of employment. The report further adds that the presence of such gaps hinders the potential for sustainable economic expansion inside the BRICS countries. The five nations were noted to have had varying degrees of advancement in resolving matters pertaining to gender equality, women's empowerment, and the protection of women's rights.

[16] also puts forward that there is an absence of innovative financing that enables women to lessen their carbon footprint and encourage soil health and water quality in the BRICS [17]. also highlights that the attainment of sustainable agricultural practices in the BRICS nations necessitates the simultaneous consideration of gender and income disparities, as well as economic prospects, both within and within these countries. Thus, failure is characterised by various expressions, such as environmental degradation, food insecurity and undernourishment, restricted opportunities for education and access to essential services, as well as the marginalisation of vulnerable groups (women, individuals with disabilities, and young people) from participating in decision-making processes.

Therefore, the primary aim of this study is to examine the potential influence of women's status on carbon emissions within the BRICS countries. The study used three indicators to measure the status of women, namely the women's political empowerment index, the women's civil liberties index and women as government chief executives. Hence, the specific inquiries which this article attempts to address are: To what extent does the political empowerment of women impact carbon emissions among the BRICS nations? To what extent do the civil liberties of women impact carbon emissions among the BRICS nations? To what extent do female government top executives influence carbon emissions within the BRICS nations?

This work provides significant contributions to the current corpus of knowledge. Firstly, according to the author's understanding, this study represents the first paper (in relation to the existing body of research studies) to investigate the influence of women's status on carbon emissions within the BRICS countries. Second, it is evident that women experience a disproportionate impact from climate change while simultaneously playing a crucial role in the mitigation of its consequences. The female gender is disproportionately affected by climate change as a result of their social, economic, and political circumstances. Additionally, they are more prone to assuming responsibility for household energy usage and food production, both of which play a substantial role in contributing to carbon emissions (roles that must not be undermined). Therefore, this research will provide significant empirical support from the standpoint of the BRICS countries. Furthermore, several studies have indicated that augmenting the participation of women in decision-making procedures correlates with the implementation of more rigorous climate change legislation, hence leading to reduced emissions. Also, the involvement of women in the management of natural resources has been found to be correlated with improved governance of resources and more favourable outcomes in terms of conservation. This study aims to provide further evidence pertaining to the aforementioned findings from the BRICS perspective. As well, this study will expose (using three distinct proxies of women's status), from the BRICS viewpoint, if it is imperative to heighten support for existing research that emphasizes the importance of acknowledging the distinct knowledge and expertise that women hold across many scales (i.e., local, national, and worldwide) and including their perspectives in decision-making procedures to facilitate the attainment of efficient climate mitigation and adaptation measures.

Furthermore, the paper significantly benefits from a more explicit connection between the identified literature gaps (literature review) and the research objectives. By strengthening this linkage, this paper enhances its academic rigor and contribution to the field. First, the paper explicitly states how the focus on BRICS nations addresses the lack of comprehensive studies on these major carbon emitters. Second, this research clearly articulates how the investigation of political empowerment, civil liberties, and female government top executives contributes to understanding the conditional distribution of environmental quality contexts of the BRICS economies, along with causal mechanisms between women's status and carbon emissions. Third, the paper emphasizes the novelty of its approach in examining all three dimensions of women's status within a single framework. Fourth, the study highlights how the focus on emerging economies and distinct cultural contexts will provide valuable insights into the potential variations in the relationship between women's status and carbon emissions. Finally, the paper clearly delineates how it will address the dearth of research on the causal processes underlying this relationship, thereby contributing to a deeper understanding of the issue.

The subsequent portions of this work are organized as follows: Section 2 of the paper presents a comprehensive literature assessment, which is divided into three main parts. Section 3 of the paper is dedicated to discussing the model specification, data, and technique in a sequential manner. Section 4 presents and examines the findings derived from the econometric analysis. Section 5 presents the implications of the study. Lastly, Section 5 provides a conclusion, summarising the key points and emphasising the further research directions.

## 2 Literature review

### 2.1 Theoretical framework

This study is based on the environmental scarcity theory formulated by Thomas Homer-Dixon [18,19]. Thus, Homer-Dixon presented his theoretical framework in relation to decreases in the relative abundance of replenished resources [[18], [19], [20]]. According to Ref. [19], he believes that as resources become scarce, it might result in violent conflicts in the developing world due to a combination of social, economic, and political factors. Homer's model utilises a conceptual framework to explain the acquisition of social reality. This framework suggests that the causal process between environmental shortage and violent conflicts may be divided into three primary stages. The text discusses the causes of environmental scarcity, its impact on society and politics, and the emergence of many types of violent conflicts [19]. Consequently, environmental scarcity is produced through the interaction of resource deterioration, population pressure, and distributional inequities. This then gives rise to the socio-economic ramifications of scarcity, encompassing limited agricultural output, the marginalisation of less influential factions, such as women, and perhaps the relocation of these factions into environmentally vulnerable regions. Thomas Homer-Dixon examines the connection between environmental deterioration, the competition for resources, and conflict and human insecurity [18,19,21]. The author advises against making the assumption that there is a direct correlation between deterioration and conflict.

Homer-Dixon categorises resource depletion and degradation into three types: supply-induced, demand-induced, and structural scarcity [[18], [19], [20], [21], [22]]. Supply-induced scarcity occurs when there is a reduction in the total amount of resources available for consumption. This scarcity is caused by the technologies and practices utilised in the use of the resource. Demand-induced scarcity occurs when there is a rise in the overall population and other alterations in consumption habits. Structural scarcity, the third type, arises from a significant disparity in the allocation of wealth and power within a society. This leads to certain groups receiving excessively large portions of resources while others receive portions that are insufficient to support their livelihoods [[18], [19], [20],^22^]. Research indicates that women face significant disadvantages in several domains of life, including politics, social interactions, economics, technology, and the environment, particularly in emerging and developing countries [23,24]. Almost every instance of resource shortage leading to war has been influenced by structural scarcity. None of these components function independently; instead, they all interact and mutually strengthen each other in different ways [19]. cautions that environmental scarcity alone is not enough to drive significant migrations, poverty, or violence. It invariably combines with other economic, political, and social elements to generate these outcomes. The sources of supply, demand, and structural factors can individually or together contribute to the overall state of environmental scarcity. Homer Dixon uses the terms resource capture and ecological marginalisation to describe the two phenomena that result from the interplay of these sources [[18], [19], [20],^22^].

Resource capture refers to the phenomenon when a decline in the amount or quality of renewable resources aligns with a population increase, leading influential groups in society to manipulate the allocation of resources in their own favour. This can result in severe environmental shortages for disadvantaged and vulnerable people whose demands for resources are contested by these influential elites [18]. Ecological marginalisation is the result of a combination of population increase and a scarcity of productive natural resources, which leads to migrations into places that are environmentally vulnerable, such as steep highland slopes, areas in danger of desertification, and tropical rainforests [25]. The high population densities in these places are predominantly composed of women, and the lack of expertise and money to safeguard local resources leads to significant environmental degradation and persistent poverty [18]. Consequently, conflicts are very probable to significantly deteriorate if few resources combine with or intensify other conflict-related societal factors.

### 2.2 Empirical studies

Numerous scholarly investigations have been done to establish empirical evidence regarding the relationship between women's status and carbon emissions. These studies have employed various models and samples. However, despite the abundance of studies, a consensus has not yet been reached on this matter. Thus, the relationship between women's status and carbon emissions is a complex one. This empirical study primarily examines three sections that analyse the existing literature pertaining to the impact of women's political empowerment, women's civil liberties, and the presence of women in government chief executive effects on carbon emissions.

### 2.3 Women's political empowerment impact on carbon emissions

[26] used panel cointegration techniques to scrutinise the environmental impacts of women's political empowerment in 72 countries from 1971 to 2012. The study proves that in the long run, women's political empowerment decreases carbon emissions. Also [27], applied a quantitative analysis of cross-national data, with a specific emphasis on the relationship between the political standing of women and per capita CO_2_ emissions. The results reveal a negative correlation between the political status of women and per capita CO_2_ emissions in sampled countries. In congruence [10], studied countries with the highest levels of carbon intensity spanning from 2000 to 2015 and augmented that the proportion of women in parliamentary positions yields a favourable impact on environmental quality [8]. utilised an extensive dataset encompassing 194 nations throughout the period from 2007 to 2015 and demonstrates that female empowerment and environmental performance are statistically significant and positively associated. Moreover [7], study on a sample of 35 European countries shows that the presence of political stability and the empowerment of women all exhibit positive and significant effects on sustainable agricultural production. Likewise [28], study conducted on Nordic and Central European nations between 2002 and 2021 provides evidence that increasing the representation of women in parliament has reduced carbon emissions [29]. survey conducted on 169 nations from 1995 to 2017 suggests that the political empowerment of women, together with its many aspects, decreases susceptibility to the effects of climate change.

### 2.4 Women's civil liberties impact on carbon emissions

[30] used threshold regressions to investigate the impact of the manufacturing sector on an environmental pollution index in nineteen Latin American nations from 1990 to 2017. The results of our study suggest that civil liberties decrease carbon emissions. The study conducted by Ref. [31] also demonstrates that institutional promotion of gender issues plays a significant role in mitigating vulnerability to climate change. Furthermore, the study suggests that the empowerment of women not only enhances their freedom but also contributes to the overall resilience of an economy. In addition [32], investigated the relationship between democracy and sustainable development on a panel of 31 nations spanning the period from 1990 to 2016, and the outcomes illustrate that a one percent rise in sustainable development is associated with a corresponding increase in democracy (civil liberties and political rights). Other authors [33], examine the link between the civil liberties of women, their engagement in civil society, elite political participation, and the sustainability of forestry practices using a sample of 101 countries. The analysis reveals that there exists a weak positive association (although statistically insignificant) between women's involvement in civil society and the sustainable forestry initiatives. To substantiate these arguments [9], analysed a sample of 101 developing countries from 1976 to 2003 and contributes that that democracy (political rights and civil liberties) has a positive impact on environmental improvement, and the effect varies depending on the specific measure of environmental quality employed (carbon dioxide emissions, water pollution, and deforestation damage) [34]. posits that when the rights of women are restricted, the consequences go beyond women themselves. Thus, the consequences have a wide-ranging impact on every aspect of society, including the natural environment [35]. survey conducted in OECD nations has demonstrated that increased levels of women's involvement in decision-making processes have a positive impact on mitigating environmental degradation through direct consequences.

### 2.5 Women as government chief executive affect carbon emissions

[11] report that a positive association between the presence of women on corporate boards and the propensity for voluntary disclosure of climate change-related information exists based on a sample of Canadian corporations that are publicly listed, spanning the years 2008–2014. Thus [5], confirm that for China, increasing the number of female employers will have both short-term and long-term effects in mitigating CO_2_ emissions. As well [36], write that companies that have female directors and smaller board sizes are more likely to accomplish better carbon emission performance and voluntarily disclose the desired level of carbon information assessed by the CDP. In this vein [37], highlight that for the examined Italian companies, the compulsory implementation of gender quotas in Boards of Directors has a detrimental impact on greenhouse gas emissions while simultaneously having a favourable association with the quantity of recycled waste. Thus, the authors express that increased representation of women directors has an impact on the decision-making process concerning environmental matters, as women demonstrate a heightened dedication to addressing this issue. A study analysing the top 100 global energy leaders between 2018 and 2020 [38], found that female leaders play a significant role in enhancing the level of carbon disclosure. However [39], posit that companies that have female external directors are inclined to exhibit reduced levels of carbon emissions. Conversely, the impacts of abatement are less pronounced when women are appointed as inside directors [40]. research on 138 companies generates mixed results. Thus, the study validates that the inclusion of women on corporate boards has a beneficial effect on the voluntary reporting of carbon emissions and the standard of such reporting. Female CEOs and women in high-level operational management have a beneficial impact on the voluntary reporting of carbon emissions. However, the presence of female board chairpersons does not affect the disclosure of carbon emissions or its accuracy.

The analysis of existing scholarly works reveals that there is a discernible presence of research on the relationship between women's status and carbon emissions. However, it is apparent that there remain some deficiencies in the empirical literature on this subject matter. A notable deficiency in the existing body of research is to the dearth of studies specifically examining the BRICS countries, namely Brazil, Russia, India, China, and South Africa. Although there may have been some research conducted on the association between women's status and carbon emissions in individual BRICS countries, a full analysis encompassing all five nations is still lacking. The existence of this disparity is of considerable importance, given that the BRICS countries represent some of the major contributors to global greenhouse gas emissions. Moreover, an additional deficiency in the existing empirical literature pertains to the dearth of research endeavours that investigate the causal processes by which the status of women may exert influence on carbon emissions. Therefore, this research aims to address this gap. There is a pressing requirement for additional empirical investigation about the link between the women status and the emission of carbon in various contextual settings. While the majority of previous studies have primarily concentrated on developed nations, further investigation is required in order to comprehend the potential variations in this association inside emerging economies and countries characterised by distinct cultural norms.

## 3 Model and data

There are numerous and diverse factors that contribute to carbon emissions. However, our study focuses specifically on the inclusion of certain women's status variables, namely the women's political empowerment index (LnPEI), the women's civil liberties index (LnCLI), and the presence of women as government chief executives (GCX). These variables are examined while controlling for economic growth (LnGDP) and population growth (LnPOP). Hence, the subsequent equation [1] is derived.

[1] $${LnCO}_{2it}=c_{it}+\beta_{1}{LnPEI}_{it}+\beta_{2}{LnCLI}_{it}+\beta_{3}{LnGCX}_{it}+\beta_{4}{LnGDP}_{it}+\beta_{5}{LnPOP}_{it}+\varepsilon_{it}$$

The variable "*i*" represents the country, where "*i*" ranges from 1, 2, 3 to *N*. Similarly, the variable "*t*" represents the time events, where "*t*" ranges from 1, 2, 3 to *T*. The notation ${LnCO}_{2it}$ refers to the natural logarithm of the concentration of carbon dioxide of a country *i* at the end of period *t.* Additionally, the equation includes an error factor, denoted as $\varepsilon_{it}$.

### 3.1 Data

This empirical research depends on the balanced panelised data spanning from 1960 to 2022 for the BRICS countries (Brazil, Russia, India, China and South Africa). The parameters used in this paper are carbon emissions (LnCO_2_), the women's political empowerment index (LnPEI), the women's civil liberties index (LnCLI), women as government chief executive (GCX) after controlling for economic growth (LnGDP) and population growth (LnPOP). Table 1 below outlines a detailed overview of the data.

**Table 1:** Describing variable features.

Variable	Definition	Unit	Source
LnCO_2_	Carbon emissions	Metric tons per capita	Our World in Data Link: https://ourworldindata.org/
LnPEI	Women's Political Empowerment Index	Ranges from 0 to 1 (most empowered).	Our World in Data Link: https://ourworldindata.org/
LnCLI	Women's Civil Liberties Index	Ranges from 0 to 1 (most rights).	Our World in Data Link: https://ourworldindata.org/
GCX	Women is government chief executive	1 when the chief executive is a woman and 0 if not.	Our World in Data Link: https://ourworldindata.org/
LnGDP	Economic Growth	GDP per capita (constant 2015 US$)	WDI Database Link: https://data.worldbank.org/
LnPOP	Population growth	Population growth (annual %)	WDI Database Link: https://data.worldbank.org/

Brief definitions of the proxies about women's status (as the main explanatory variable):

Women's Political Empowerment Index: The index assesses the degree to which women possess and exercise civil freedoms, engage in civil society, and hold political representation. Hence, “It captures the extent to which women enjoy civil liberties, can participate in civil society, and are represented in politics ” [41].

Women's Civil Liberties Index: The index encapsulates the degree to which women experience freedom from coerced labour, possess property rights and access to the legal system, and exercise the right to unrestricted mobility. Therefore, “It captures the extent to which women are free from forced labour, have property rights and access to the justice system, and enjoy freedoms of movement” [42].

Women is government chief executive: This means the chief executive is a woman who holds the highest position of authority within a government or state, typically with greater power than other officials. Thus, “Chief executive is head of government or head of state, whoever has more power” [43].

It is imperative to that that this paper obtained the data for this analysis from the World Bank Indicators and Our World in Data databases, which are publicly accessible. This method guaranteed the research's replicability and transparency. The BRICS economies have undergone substantial environmental, financial, economic, and social transformations from 1960 to 2022. This long-term perspective enables a thorough examination of the continuously changing relationship between women's status and carbon emissions, taking into account historical trends, policy changes, and economic development. Furthermore, the incorporation of data from 1960 establishes a foundation for comprehending the origins of these relationships and their progression over time.

### 3.2 Econometric procedure

This section provides an overview of the econometric methodologies employed in the present study.

#### 3.2.1 Panel quantile regression with fixed effects

The principal econometric tool applied in this paper is the novel panel Methods of Moments Quantile Regression (MMQR) with fixed effects. It is employed to investigate the possible influences of these different proxies of women's status and adopted controlling factors on carbon emissions across the conditional distribution of, indeed, environmental quality levels of the BRICS economies. Considering works by Refs. [44,45], the conditional quantile of a random factor $Q_{Y}$ (*τ*|X) is illustrated by the following:

[2] $$Y_{it}=\beta_{i}+X_{it}^{\prime }\alpha +\left(\theta_{i}+Z_{it}^{\prime }\Phi \right)\;\Psi_{it}$$

In this regard, $Y_{it}$ is the response factor, $X_{it}$ demonstrates the independent and identically distributed endogenous parameters (β, α, Φ, Ψ) are variables to be evaluated. The likelihood, P {$\theta_{i}$ + $Z_{it}^{\prime }\Phi$ > 0} = 1. Meeting moment conditions, $\Psi_{it}$ refers to independent and identically unobserved random factors distributed across individuals and orthogonal to $X_{it}$ [46]. *i* = 1, 2, 3 …. *N* illustrates the individual *i* fixed effects. *Z* is a k-vector noted parts of *X.*

Taking into account [44,45], equation [2] evolves into equation [3] as follows:

[3] $$Q_{Y}\left(\tau |X_{\text{it}}\right)=(\beta_{i}+\theta_{i}\;q\left(\tau \right))+X_{it}^{\prime }\alpha +Z_{it}^{\prime }\Phi \;q\left(\tau \right)$$

In this setting, the response parameter, $Y_{it}$ has $Q_{Y}$ (*τ*| $X_{\text{it}}$) as its quantile distribution. $\beta_{i}$ (*τ*) ≡ $\beta_{i}$ + $\varepsilon_{i}$ q(*τ*) depicts the scalar estimate, while the sample quantile is identified by *τ*^th^ [46]. *Z* is a k-vector noted parts of $X_{it}$ normalised to meet moment conditions, E(Ψ) = 0 and E(I Ψ I) = 1.

Therefore, the MMQR model of this research includes all parameters of this study. Thus, alternatively, equation [3] may be expressed as equation [4], which is shown as follows:

[4] $${QLnCO}_{2it}\left(\tau_{k}\;|\beta_{i},X_{\text{it}}\right)=c_{it}+\beta_{1\tau }{LnPEI}_{it}+\beta_{2\tau }{LnCLI}_{it}+\beta_{3\tau }{GCX}_{it}+\beta_{4\tau }{LnGDP}_{it}+\beta_{5\tau }{LnPOP}_{it}$$

It is vital to note that this research was determined to implement the Moments Quantile Regression Method due to its numerous benefits. Initially, it enables a more sophisticated examination of the relationship between women's status and carbon emissions by analysing various quantiles of the conditional distribution, thereby offering a better understanding of how this relationship varies across varying levels of carbon emissions. Secondly, it is resilient to outliers and heteroscedasticity, which are prevalent in economic and environmental data. Third, it is more adaptable than conventional regression methods in that it does not necessitate assumptions regarding the functional form of the relationship. Fourth, it is computationally efficient and capable of managing large datasets, rendering it appropriate for the analysis of the BRICS economies. Lastly, the study's methodological innovation is enhanced by the implementation of the Method of Moments Quantile Regression, which provides a unique and novel method of investigating the gender-environment nexus.

#### 3.2.2 Panel causality test

This research utilises a heterogeneous non-causality test [47] to analyse casuality involving the variables included in the panel. The panel data possess a very large T dimension (63 years) compared to the N dimension (5 economies); hence, the deployment of asymptotic distributions is proven. This approach is widely accepted because the Monte Carlo simulation developed by this technique is appropriate even in cases where cross-sectional dependency is existent. As such, the cross-sectional dependency outcome does not considerably influence the rigorousness of the estimates. Moreover, this model is founded on a vector autoregressive model and is appropriate for a balanced heterogeneous dataset. The regression proposed by Ref. [47] for identifying causality in panel data is demonstrated in equation [5]. In this vein, a panelised linear design, which features a pair-up of parameters, $y_{it}$ and $x_{it}$, for instance, is highlighted as follows:

[5] $$y_{i,t}=\alpha_{i}+\sum_{r=1}^{R}\gamma_{i}^{\left(k\right)}y_{i,t-r}+\sum_{r=1}^{R}\beta_{i}^{\left(k\right)}x_{i,t-r}+\varepsilon_{i,t},i=1,2,\ldots \ldots .N:t=1,2,\ldots ..,T$$

It follows that the lag length is *k*, and the autoregressive variable is depicted as γ^(k)^, and β^(k)^ represents the equation estimate, which is flexible within the group with a normal, normal, independent and identically distributed error term denoted as $\varepsilon_{i,t}$ for a particular cross-section (*i*) and time (*t*).

As regards to this test, the null hypothesis, depicted as Homogenous Non-Causality (HNC) hypothesis, is presented as follows: $H_{o}$: $\beta_{i}$ = 0, ∀i=1,…….N. On the other hand, the alternative hypothesis, recognised as the Heterogeneous Non-Causality (HENC) hypothesis, alluding to the existence of a causal relationship in at least one cross-section, is given as follows:

$$H_{1}:\beta_{i}=0,\forall i=1,\ldots \ldots .N_{1}$$

$$\beta_{i}\neq 0,\forall i=N_{1}+1,\ldots \ldots \;N$$

#### 3.2.3 Cross-section dependence test

In relation to the process of panel data analysis, it is necessary to first examine the framework in order to determine the presence or absence of cross-sectional dependency before assessing the stationarity of the series. Consequently, the hypotheses formulated to examine cross-sectional dependence are outlined as follows:

$H_{o}$: Cross-sectional independence.
$H_{1}$: No cross-sectional independence.

In the event that the null hypothesis $H_{o}$ is rejected, a first-generation unit root test will be utilised. Conversely, if the null hypothesis $H_{o}$ is accepted, a second-generation unit root test procedure will be implemented. This test is vital in light of the fact that neglecting the cross-sectional dependence (CD) test might lead to biased outcomes, distorted sizes, and inconsistent results. Hence, it is crucial to verify the presence of CD. We employed the CD test devised by Ref. [48] to evaluate the interdependence of the diverse macroeconomic variables, as seen in equation [6]. The null hypothesis posits the absence of class dependency, whereas the alternative hypothesis asserts the presence of cross-sectional dependence among the variables. The following test statistics are shown in equation [6] as follows:

[6] $$\text{CD}=\surd \frac{2T}{N\left(N-1\right)}\sum_{i=1}^{N-1}x\;\sum_{i=1}^{N}x\;\rho_{ij}$$

In this case, the symbol $\rho_{ij}$ represents the pairwise correlation. However, notwithstanding the existence of a causal relationship between the nations, it is important to acknowledge that each country can also preserve its own unique dynamic. Assuming a uniform slope coefficient in this context might lead to erroneous and misleading outcomes [49]. Consequently, it is crucial to conduct a hypothesis test to evaluate the null hypothesis of equal slopes. In the context of normally distributed error parameters, the bias adjustment of the mean variance is denoted by $\tilde{\Delta }adj$ as shown in equation [7]. Thus, the test is conducted by:

[7] $$\tilde{\Delta }adj=\sqrt{N\;\frac{S{\tilde{z}}_{it})\;N^{-1}}{\sqrt{var(\tilde{S}}\;{\tilde{z}}_{it})}}$$

In this regard, *E*(${\tilde{z}}_{it}$) is equal to *k*, and the denoted variance, var (${\tilde{z}}_{it}$) is equal to *2k(T-k-1/T* + *1*).

The result of the analysis using first-generation CDs will enable the research to determine the series order of integration using second-generation panel unit techniques. Hence, this research utilises the CIPS tests to examine the stationarity of the economies among the BRICS nations. The CIPS test, as articulated by Ref. [50], may be assessed by calculating the mean of the t-statistics for a specific cross-sectional unit within a selection of the BRICS member economies, as demonstrated in equation [8]. Hence, the equation for the CIPS test [50] can be expressed as:

[8] $$\text{CIPS}\;\left(N,T\right)=N^{-1}\;\sum_{i=1}^{N}t_{i}\left(N,T\right)$$

#### 3.2.4 Co-integration approaches

This paper will also examine if the data exhibit co-integration by employing heterogeneous panel co-integration methodology as proposed by Refs. [51,52]. The co-integration technique developed by Refs. [51,52] is founded on residuals that consider the variations in specific impacts, slope estimations, and individual linear patterns across countries [52]. proposed the development of two variations of residual-oriented tests. The first set of tests, namely the panel v-statistic, panel rho-statistic, panel PP-statistic, and panel ADF-statistic, are four often used tests that follow a conventional normal distribution in the asymptotic limit. These tests are based on pooling residuals inside each category of the within-dimension. Regarding the second statistic, namely the group rho-statistic, group PP-statistic, and the group ADF-statistic, it is important to note that these statistics are structured in a manner that conforms to the usual normal distribution in an asymptotic sense. Additionally, it is worth mentioning that these statistics are based on the practice of pooling residuals for classification purposes across several dimensions. These statistical methods often involve the calculation of the mean of individually computed coefficients for each member. They take into account specific short-term changes, fixed impacts, and deterministic directions, as well as estimated coefficients for slopes [53]. [52,53] employs Monte Carlo simulations to establish that the panel ADF-statistic and group ADF-statistic tests exhibit superior reliability in comparison to alternative tests, as they possess more favourable small sample characteristics.

## 4 Results and discussion

Table 2 shows important statistical information. As an illustration, the arithmetic average of economic growth is the highest and determined to be 3.089. This indicates that the collective economic growth for all countries included in the table has an average value of 3.089. The standard deviation quantifies the dispersion or variability of data points within a dataset. For instance, the computed standard deviation of carbon emissions is 0.5077, indicating that the dataset exhibits dispersion across a range of 0.5077 units both above and below the mean. The skewness value for women as government executives (GCX) exceeds 3, signifying the highest level of skewness and showing that the variable is highly skewed towards the right. This implies that the distribution has a positively skewed pattern, characterised by a small number of exceptionally big values in the right tail. The variables women's political empowerment, economic growth, population growth, and women as government executives exhibit a kurtosis value over 3, suggesting that they possess a leptokurtic distribution characterised by a more pronounced peak and heavier tails compared to a normal distribution. This implies that there is a greater presence of outliers in both the positive and negative directions.

**Table 2:** Presents the statistical information pertaining to the variables.

Statistics	LnCO_2_	LnPEI	LnCLI	LnGDP	LnPOP	GCX
Min	−0.6027	−0.6696	−1.4437	0	−1.5213	0
Std.Dev	0.5077	0.1627	0.3934	1.1057	0.3249	0.2442
Max.	1.2337	−0.0462	−0.0851	4.0630	0.5438	1
Mean	0.4589	−0.2595	−0.4558	3.0890	0.0922	0.0635
Skewness	−0.2277	−1.1756	−1.0433	−1.8938	−1.5986	3.5802
Kurtosis	1.7894	3.8307	2.9164	5.7632	7.1343	13.8178

**Fig. 1:**
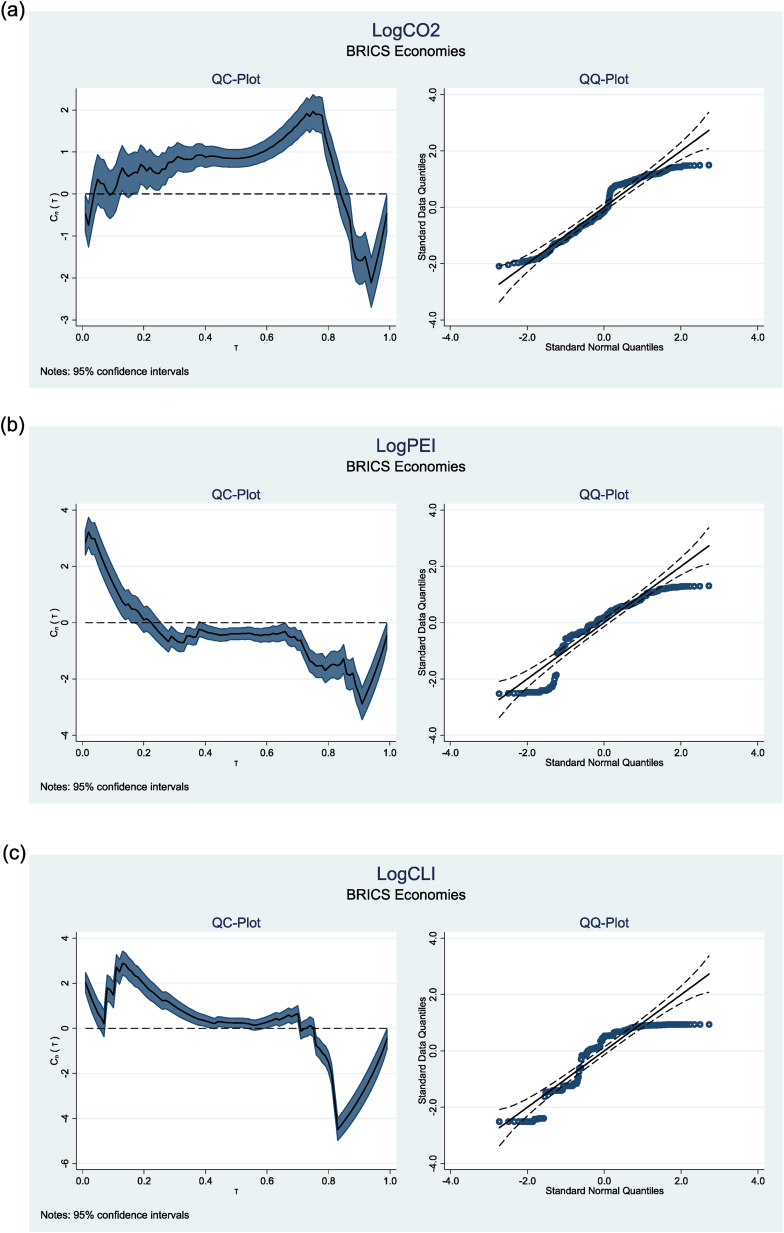

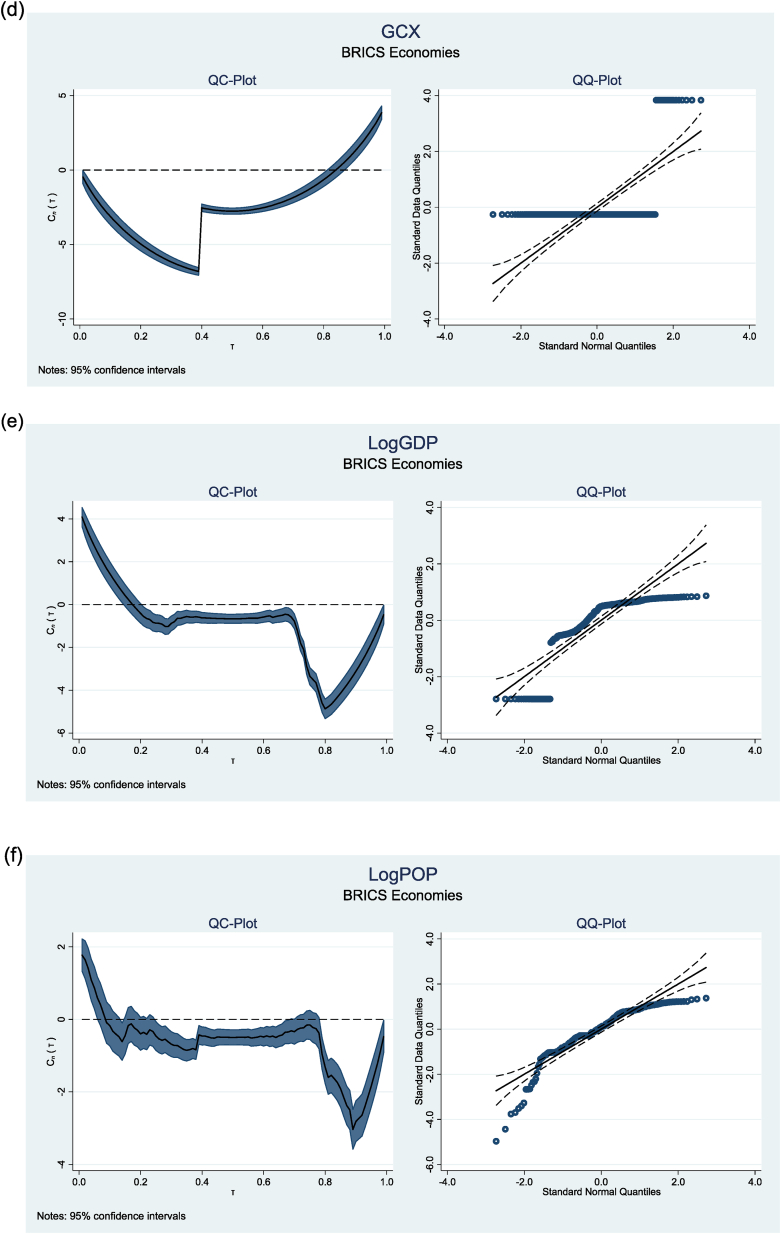
Showing Q-Q plots and Q-C plots of variables

This study also performed a normality test utilising quantile mean covariance [54]. The results are illustrated in Fig. 1(a–f). The QC plots exhibit deviations from the horizontal line at the extreme quantiles, indicating non-normal distributions. Furthermore, the Q-Q plots corroborate significant departures from the horizontal line, raising concerns about the data's normality. These results validate the application of the MMQR methodology.

As indicated in Table 3, all variables, with the exception of GCX, exhibit a p-value of 0.0000 for their Pesaran [53] cross-section dependent test values. This finding suggests that the null hypothesis of cross-sectional independence can be rejected at a significance level of 1 % for the variables. Hence, there exists robust statistical evidence indicating a significant cross-sectional dependence among the metrics of the BRICS countries, except for GCX, implying a correlation among them. Moreover, the study refutes the notion that slope estimations exhibit homogeneity throughout the cross-sections, regardless of the significance levels. This implies the presence of heterogeneous slopes in the main equation. The following section of the study examines the outcomes of the second-generation unit root tests- CIPS.

**Table 3:** Depicts Pesaran CD test findings and slope homogeneity results.

Variables	CD_Pesaran_ test (2004)	
	Statistic	*p*-value
LnCO_2_	12.62	0.0000∗∗∗
LnPEI	22.45	0.0000∗∗∗
LnCLI	21.29	0.0000∗∗∗
GCX	–	–
LnGDP	18.89	0.0000∗∗∗
LnPOP	14.87	0.0000∗∗∗
**Slope-homogeneity – with LogCO**_**2**_**as the dependent parameter**		
Delta	27.549 ∗∗∗	
Adj. Delta	29.220 ∗∗∗	

Notes [1]: ∗∗∗; ∗∗ and ∗ indicate that the coefficients are significant at the 1 %, 5 % and 10 % level of significance, respectively.

The findings in Table 4 outline that all parameters, except GCX, can reject the null hypothesis of a unit root at their first difference since their differenced p-value is less than all three critical values. This means that the variables, except GCX, are stationary at the first difference. The next part of the research illustrates the cointegration results of the paper.

**Table 4:** The results of the CIPS tests.

Variables	Level [constant and trend]	First Difference [constant and trend]
LnCO_2_	−2.832	−5.639
LnPEI	−1.974	−5.972
LnCLI	−2.194	−5.674
GCX	−0.172	−1.548
LnGDP	−2.186	−5.908
LnPOP	−4.501	−6.420

Notes [1]: The critical values at 1 %, 5 % and 10 % are −2.72, −2.83 and −3.04 respectively.

Table 5 presents the results of the Pedroni cointegration tests. Therefore, based on the results of 6 out of 11 tests, it can be concluded that the p-values obtained are statistically significant at the 1 % and 5 % levels of significance. This suggests that the null hypothesis, which assumes no cointegration, can be rejected at the 1 % and/or 5 % significance level. Hence, substantial statistical evidence exists to indicate the presence of a cointegrating link among the variables in the panel regression. A cointegrating link between two or more variables implies that these variables exhibit a long-term association, notwithstanding the possibility of short-term fluctuations. The significance of this observation comes in its implication of the existence of a stable equilibrium relationship between the variables in the long term.

**Table 5:** The results of the Pedroni panel co-integration tests.

Pedroni panel co-integration tests – Findings					
Within-dimension (panel statistics)			Between-dimension (panel statistics		
**Test**	**Statistic**	**Probability**	**Test**	**Statistic**	**Probability**
**Pedroni (1999)**					
Panel v-statistic	6.6664	0.000∗∗∗	Panel rho-statistic	−2.5689	0.0051∗∗∗
Panel rho-statistic	−2.7834	0.0027∗∗∗	Panel PP-statistic	−1.1415	0.1268
Panel PP-statistic	−1.1138	0.1327	Panel ADF-statistic	−0.3593	0.3597
Panel ADF-statistic	−0.6440	0.2598			
**Pedroni (2004) Weighted Statistic**					
Panel v-statistic	6.249	0.0000∗∗∗			
Panel rho-statistic	−3.2294	0.0006∗∗∗			
Panel PP-statistic	−1.6929	0.0452∗∗			
Panel ADF-statistic	−1.1120	0.1331			

Notes: ∗∗∗: ∗∗ and ∗ indicate that the coefficients are significant at the 1 %, 5 % and 10 % level of significance, respectively.

Table 6 presents the results of the Method of Moments Quantile regression analysis, showcasing the predicted variables across several quantiles ranging from 10 % to 90 %. To begin, the table presents the coefficients pertaining to the independent variable, LnPEI- women's political empowerment index. Notably, all of these coefficients exhibit a negative sign. Furthermore, as the quantile increases, the coefficients demonstrate a greater degree of statistical negativity. This implies an inverse relationship between carbon emissions and women's political empowerment, whereby higher values of the variable correspond to lower levels of carbon emissions for the BRICS. Likewise [26], findings derived from the pooled mean group (PMG) estimator demonstrate that, in the long term, there is a reduction of around 11.51 percentage points in CO_2_ emissions in response to a one-unit rise in the Women's political empowerment index, while accounting for various other factors.

**Table 6:** Quantile regression estimates for BRICS countries.

Quantile Var	Location	Scale	$Q_{0.10}$	$Q_{0.20}$	$Q_{0.30}$	$Q_{0.40}$	$Q_{0.50}$	$Q_{0.60}$	$Q_{0.70}$	$Q_{0.80}$	$Q_{0.90}$
LnPEI	−0.229	−0.113	−0.041	−0.127	−0.168	−0.213	−0.238∗	−0.28∗∗	−0.302∗∗	−0.340∗∗∗	−0.405∗∗
LnCLI	0.252∗∗∗	−0.098	0.415∗∗	0.341∗∗	0.305∗∗∗	0.266∗∗∗	0.244∗∗∗	0.208∗∗	0.190∗∗	0.156∗	0.100
GCX	−0.178∗∗∗	0.013	−0.199∗	−0.189∗∗	−0.185∗∗	−0.179∗∗∗	−0.177∗∗∗	−0.172∗∗∗	−0.169∗∗∗	−0.165∗∗∗	−0.158∗∗
LnGDP	−0.021	0.015	−0.056	−0.044	−0.038	−0.032	−0.029	−0.023	−0.020	−0.015	−0.006
LnPOP	−0.316∗∗∗	−0.287∗∗∗	0.161	−0.055	−0.161	−0.275∗∗	−0.338∗∗∗	−0.443∗∗∗	−0.498	−0.60∗∗∗	−0.759∗∗∗

Notes [1]: ∗, ∗∗ and ∗∗∗significant at 1, 5 and 10 % levels of significance.

As well, the analysis shown in Table 6 reveals a positive and statistically significant relationship between the women's civil liberties index (LnCLI) and carbon emissions across the majority of quantiles. This suggests that, in general, a one percent increase in women's civil liberties is significantly contributing to elevated emissions. However [27], emphasised on the relationship between the political standing of women and per capita CO_2_ emissions by a cross-country analysis (as they postulated that cultures characterised by higher levels of gender equality are likely to exhibit comparatively reduced environmental consequences). Their results show that countries with a greater political status for women tend to exhibit lower per capita CO_2_ emissions.

Furthermore, the data shown in Table 6 proves that across various quantiles, there is a negative and statistically significant relationship between the presence of women as government chief executives (GCX) and environmental quality. This relationship demonstrates a very high level of strength in the upper quantiles. There exist several potential explanations for this observed association. One potential explanation posits that women in these countries exhibit a higher propensity to prioritise environmental concerns compared to men. An alternative perspective posits that women in the BRICS exhibit a higher propensity to endorse and implement initiatives that provide favourable outcomes for the environment. Thirdly, there exists a possibility that BRICS countries with pre-existing high levels of environmental quality are more inclined to elect women to positions of power.

In agreement with this research findings [55], posit that there is a positive correlation between the proficiency of chief executive officers (CEOs) and the disclosure of corporate environmental sustainability information, and the presence of a female CEO enhances the association between the competence of the CEO and the sharing of information pertaining to business environmental sustainability. In the same vein [56], also highlight that there is a negative correlation between the proportion of female directors and carbon emissions, implying that as the percentage of female directors increases, carbon emissions decrease.

For the controlling variables, it is proven that economic growth develops a negative, non-statistically significant relationship with carbon emissions across all quantiles. This relationship may be explained in a number of different ways. One plausible perspective posits that BRICS nations exhibiting higher rates of economic growth are inclined to allocate resources towards the development and implementation of renewable energy sources and energy efficiency initiatives. An alternative viewpoint posits that BRICS nations experiencing more economic growth are more inclined to adopt laws aimed at mitigating carbon emissions. Howbeit, disagreeing with this paper's results [57], add that, for Chile, economic growth has been observed to contribute to an increase in consumption-based carbon emissions. In addition [58], confirm that, for Turkey, an increase of 1 % in economic growth would correspondingly result in a rise of 0.39 % carbon dioxide emissions.

In addition, population growth association with carbon emissions is negatively significant in most quantiles, especially in the upper levels. This finding also informs that the link between population growth and emissions is strongest in BRICS countries with higher populations (for example, they are more industrialised and hence generate more emissions, or their greater population consumes more resources, leading to higher emissions). However, for the United States during the period spanning from 1971 to 2016 [59], demonstrated that population growth has a positive association with carbon emissions such that this variable leads to the degradation of environmental quality. Likewise [60], observes that an increase in population growth has a positive correlation with CO_2_ emissions.

The next section presents the causality test findings.

The causality test results between various variables and emissions are presented in Table 7, as conducted by Dumitrescu and Hurlin. The Dumitrescu and Hurlin test is a panel data causality test that exhibits greater resilience to cross-sectional dependence and serial correlation compared to conventional Granger causality tests. Firstly, the results show an uni-directional casuality from the women's political empowerment index (LnPEI) to carbon emissions. This implies that a causal association exists between women's political empowerment and carbon emissions, albeit unidirectional in nature. Put, women's political empowerment has the potential to influence alterations in carbon emissions. However, carbon emissions do not possess the capacity to induce modifications in women's political empowerment. In this regard, we could highlight that BRICS countries characterised by elevated levels of women's political empowerment are inclined to pursue policies that yield favourable outcomes for the environment. For instance, these nations may have a higher propensity to allocate resources towards the adoption and implementation of environmentally sustainable methods. At the same time, we can also explain that women illustrate a higher tendency for expressing worry regarding climate change and other environmental matters, potentially leading to a greater likelihood of voting for candidates who advocate for environmental preservation.

**Table 7:** Showing Causality test results between variables and emissions.

Null hypothesis	Statistic	Prob	Causality Flow
LnCO_2_ does not homogenously cause LnPEI	−0.0905	0.9279	LnPEI → LnCO_2_
LogPEI does not homogenously cause LogCO_2_	1.7655	0.0775∗	
LnCO_2_ does not homogenously cause LnCLI	4.9262	0.000∗∗∗	LnCO_2_ → LnCLI
LnCLI does not homogenously cause LnCO_2_	0.3221	0.7474	
LnCO_2_ does not homogenously cause LnGDP	3.1299	0.0017∗∗∗	LnCO_2_ → LnGDP
LnGDP does not homogenously cause LnCO_2_	0.7286	0.4662	
LnCO_2_ does not homogenously cause GCX	0	0	–
GCX does not homogenously cause LnCO_2_	0	0	
LnCO_2_ does not homogenously cause LnPOP	28.5120	0.000∗∗∗	LnPOP ↔ LnCO_2_
LnPOP does not homogenously cause LnCO_2_	14.2613	0.000∗∗∗	

Notes: ∗∗∗; ∗∗ and ∗ indicate that the coefficients are significant at the 1 %, 5 % and 10 % level of significance, respectively.

Second, the results illustrate that a one-way casuality is demonstrated from carbon emissions to the women's civil liberties index (LnCLI). This signifies that carbon emissions and women's civil liberties are causally related, albeit in a unidirectional fashion. To clarify, it can be stated that carbon emissions have the potential to induce alterations in women's civil liberties. However, it is not plausible for the women's civil liberties to instigate variations in carbon emissions. Several potential reasons exist for this observed association. One potential explanation posits that the release of carbon emissions can potentially result in adverse economic consequences, thereby contributing to a deterioration in the civil rights of women. In BRICS nations significantly impacted by climate change, it is plausible that women face a heightened risk of displacement from their residences, have challenges related to food availability, and become vulnerable to acts of violence. An alternative scenario involves the potential for carbon emissions to contribute to a deterioration in the overall standard of living, subsequently resulting in a reduction of women's civil liberties. In BRICS nations characterised by elevated levels of air pollution, there exists a heightened probability for women to have adverse health conditions, hence impeding their capacity to engage in societal activities and exercise their entitlements.

Third, the findings in Table 7 denote the presence of an uni-directional casuality from economic growth (LnGDP) to carbon emissions. The rationale behind this phenomenon is the fact that economic expansion is frequently propelled by carbon-emitting activities, including the utilisation of fossil fuels and the non-green manufacturing of commodities and services. As an economy experiences growth, there is an increased tendency for individuals to possess and operate automobiles, provide heating for their residential and commercial spaces, and engage in heightened consumption of products and services. All of these activities are associated with the generation of carbon emissions.

Moreover, a bi-directional causality association is noted between population growth and carbon emissions. This indicates that the two variables are causally related in both directions. Hence, population expansion can lead to alterations in carbon emissions, whereas carbon emissions can also result in variations in population increase in the BRICS. There exist several potential explanations for this reciprocal interaction. The paper can possibly spotlight that the expansion of population size engenders a corresponding surge in the need for commodities and services, thus resulting in an upsurge in carbon emissions. As the population expands, there is an increased need for housing, food, and transportation, leading to a heightened generation of carbon emissions. The study can also highlight that the emission of carbon can potentially result in a deterioration of overall health and well-being, therefore leading to a decrease in fertility rates. One illustrative instance involves the potential consequences of being exposed to air pollution, which might manifest in respiratory complications and several other health-related issues. This phenomenon can potentially provide challenges for couples in their attempts to conceive and procreate.

## 5 Implications of the study

The implications of an inverse relationship between carbon emissions and women's political empowerment are significant. It suggests that countries with higher levels of women's political empowerment tend to have lower levels of carbon emissions. Moreover, a one-way casuality link is evident from women's political empowerment to carbon emissions. Thus, this paper proposes that the political empowerment of women is among the most efficacious strategies for mitigating carbon emissions. Furthermore, it suggests that climate policy has to be formulated in a manner that fosters gender equality. For instance, it is imperative for climate policy to prioritise the promotion of women's leadership within the clean energy industry while also facilitating their enhanced access to resources and opportunities within the green economy.

This study additionally observes a substantial and positive relationship between women's civil liberties and carbon emissions across a majority of quantiles. Therefore, ensuring climate justice and gender equality is crucial in order to promote women's empowerment and economic development in a sustainable fashion. This entails guaranteeing equal access to a healthy and sustainable environment for individuals, irrespective of their gender. Furthermore, it is imperative for BRICS countries with elevated levels of women's civil liberties to oversee economic progress through the implementation of environmentally friendly strategies and policies. This is because non-green scenarios are linked to heightened consumption and energy utilisation by women, which in turn can result in amplified carbon emissions. Furthermore, it is imperative to enhance green educational activities within the labour force due to the growing opportunities for women to pursue higher education and seek employment in contemporary society.

Furthermore, there is evidence of a unidirectional causal relationship from carbon emissions to the women's civil liberties in this paper. As such, high carbon emissions have the potential to cause social unrest and conflict, force women to relocate to areas with fewer rights and opportunities, make it more difficult for women to access basic services (like health and education), and increase gender-based violence. For these reasons, the BRICS should be very concerned about this outcome. In light of this, it is imperative for the BRICS nations to allocate resources towards implementing adaptation strategies aimed at assisting populations in effectively managing the consequences of climate change, including but not limited to rising sea levels, severe weather occurrences, and changes in agricultural productivity. Furthermore, it is imperative to provide assistance to environmental organisations and movements that specifically focus on women's issues.

This paper also found that there is a negative and statistically significant relationship between the presence of women as government chief executives (GCX) and carbon emissions. Consequently, if the goals of sustainable development are to be achieved, it is critical that senior leadership positions in national institutions be filled with women. This discovery suggests that female government top executives may have a greater inclination to attentively consider the demands of their constituents and formulate policies that align with those demands. This may encompass policies that facilitate the advancement of environmental sustainability, alongside policies that tackle additional social and economic dilemmas. Furthermore, these women have the capability to establish confidence and foster cooperation with other nations regarding matters pertaining to climate change. This has the potential to expedite the rate of international efforts in addressing climate change. Additionally, this study posits that female government chief executives are urged to serve as role models, inspiring other women and girls to participate in the realms of politics and environmental activism actively. This has the potential to foster a broader range of perspectives and promote inclusivity within the political sphere, so yielding advantages for both democratic governance and environmental sustainability.

This analysis additionally demonstrates that there exists a negative association between economic growth and carbon emissions, albeit one that lacks statistical significance across all quantiles. This paper also indicates the existence of unidirectional causality from economic growth to carbon emissions. This implies that it is possible for the BRICS nations to attain economic expansion while minimising environmental damage. This situation presents a mutually beneficial outcome for both the economy and the environment. In this context, the BRICS countries are potentially enhancing their energy efficiency, making investments in renewable energy sources, implementing sustainable land use and forestry practices, and adopting strategic green policies to foster sustainable growth. It is imperative for the BRICS nations to prioritise the attainment of their climate objectives while ensuring sustained economic growth. This entails persistently investing in renewable energy sources, fostering the growth of environmentally friendly industries and services, and implementing measures that enhance air quality and subsequently reduce emissions.

This study also demonstrates a statistically significant negative relationship between population growth and carbon emissions across various quantiles, particularly at higher levels. The significantly negative link involving population growth and carbon emissions, particularly at higher quantile levels, holds considerable consequences. This indicates that nations characterised by lower rates of population growth generally exhibit correspondingly lower levels of carbon emissions, particularly within the subset of the BRICS countries known for their high levels of emissions. Therefore, it is plausible that this association can be attributed to several contributing factors. Hence, it can be observed that BRICS nations characterised by lower rates of population increase exhibit a tendency towards having older demographic profiles. Elderly individuals typically exhibit lower energy consumption rates and possess smaller carbon footprints in comparison to their younger counterparts [61]. It might be posited that BRICS countries attributed to lower rates of population increase exhibit a higher degree of development. Developed nations often exhibit higher levels of economic efficiency and possess energy systems denoted by greater environmental sustainability compared to their developing counterparts. Furthermore, it can be inferred that BRICS countries designated by lower rates of population growth exhibit more robust environmental legislation and institutions. Addressing climate change can be significantly facilitated by implementing measures to reduce population growth in the BRICS countries. Therefore, it is imperative for the BRICS countries to synchronise their efforts in attaining their climate objectives by taking into account population dynamics, such as implementing measures related to family planning in conjunction with enhancing public health and overall well-being.

## 6 Conclusion

Environmental pollution is still increasing worldwide despite a number of empirical research, international platform activities, and other measures. Thus, an investigation of the impact of various elements related to women's status on environmental degradation is a crucial endeavour in the context of our continuously changing planet. This study examined the influence of the women's political empowerment index, women's civil liberties index, and the presence of women as government chief executives on carbon emissions in the BRICS economies from 1960 to 2022. The study employed the novel Method of Moments Quantile regression and the Dumitrescu and Hurlin causality test to analyse the sampled data.

The paper's findings demonstrate a statistically significant inverse relationship between carbon emissions and women's political empowerment. Furthermore, there is a clear unidirectional causal relationship observed between the empowerment of women in politics and carbon emissions. This study additionally discovered a strong and inverse link between the representation of women in positions of government chief executives and carbon emissions. As well, the paper further finds a significant and positive association between women's civil liberties and carbon emissions over a majority of quantiles. Moreover, this research presents evidence supporting a unidirectional causal link between carbon emissions and women's civil liberties. This analysis further illustrates the presence of a negative connection between economic growth and carbon emissions; however, this relationship does not exhibit statistical significance across all quantiles. This paper also suggests the presence of a one-way causal relationship from economic growth to carbon emissions. This study additionally presents empirical evidence of a statistically significant inverse relationship between population growth and carbon emissions across multiple quantiles, with a more pronounced effect shown at higher quantile levels. Furthermore, it is observed that there exists a reciprocal causal relationship between population growth and carbon emissions.

This study yields intriguing results, yet it is crucial for this research to acknowledge certain potential limitations. This research only examines the BRICS economies, namely Brazil, Russia, India, China, and South Africa. Therefore, the generalizability of the findings to other nations or areas may be restricted. Furthermore, although the time span from 1960 to 2022 is considerable, some elements of women's societal standing or environmental restrictions may undergo substantial transformations throughout this period. Moreover, the study may fail to include other crucial elements that influence carbon emissions, such as technology improvements, energy legislation, or industrial architectures. As well, the research also centres its attention on the enhancement of women's political influence and the development of their effective leadership. However, additional factors such as women's education, involvement in the workforce, and access to resources may also have an influence and might be investigated in future studies. Recognising these constraints enhances the study and facilitates future research that enhances the comprehension of the intricate correlation between women's status and environmental quality.

In various contexts, further research can be conducted on the association between the status of women and carbon emissions. The research primarily centres on the BRICS nations; nonetheless, it is crucial to comprehend the potential variations in this relationship between countries with disparate levels of development and distinct cultural norms. Furthermore, further study is required to have a comprehensive understanding of the strategies and initiatives that may be formulated to enhance the societal standing of women and mitigate carbon emissions. The research offers several recommendations, amongst others, for allocating resources towards women's green education and empowerment in the field of environmental sustainability, as well as for the promotion of women to leadership roles. However, further investigation is necessary to formulate and assess the efficacy of policies and initiatives in this regard. Further research could focus on investigating the interplay between gender, climate change, and various socio-economic factors, including poverty, inequality, and war. Examining the relationship between women's status and several environmental indicators, including air and water quality, as well as biodiversity, holds immense significance.

## Data availability

Data will be made available on request.

## Funding statement

This research did not receive any specific grant from funding agencies in the public, commercial, or not-for-profit sectors.

## Declaration of Competing Interest

The authors declare that they have no known competing financial interests or personal relationships that could have appeared to influence the work reported in this paper.
